# Mechanism of Cellular Oxidation Stress Induced by Asymmetric Dimethylarginine

**DOI:** 10.3390/ijms13067521

**Published:** 2012-06-18

**Authors:** Srinidi Mohan, Ho-Leung Fung

**Affiliations:** 1 Department of Pharmaceutical Sciences, University of New England, Portland, ME 04103, USA; E-Mail: smohan@une.edu; 2 Department of Pharmaceutical Sciences, University at Buffalo, State University of New York, Buffalo, NY 14260, USA

**Keywords:** asymmetric dimethylarginine, tetrahydrobiopterin, endothelial nitric oxide synthase, oxidative stress

## Abstract

The mechanism by which asymmetric dimethylarginine (ADMA) induces vascular oxidative stress is not well understood. In this study, we utilized human umbilical vein endothelial cells (HUVEC) to examine the roles of ADMA cellular transport and the uncoupling of endothelial nitric oxide synthase (eNOS) in contributing to this phenomenon. Dihydroethidium (DHE) fluorescence was used as an index of oxidative stress. Whole cells and their isolated membrane fractions exhibited measureable increased DHE fluorescence at ADMA concentrations greater than 10 μM. ADMA-induced DHE fluorescence was inhibited by co-incubation with L-lysine, tetrahydrobiopterin (BH_4_), or L-nitroarginine methyl ester (L-NAME). Oxidative stress induced in these cells by angiotensin II (Ang II) were unaffected by the same concentrations of L-lysine, L-NAME and BH_4_. ADMA-induced reduction in cellular nitrite or nitrite/nitrate production was reversed in the presence of increasing concentrations of BH_4_. These results suggest that ADMA-induced DHE fluorescence involves the participation of both the cationic transport system in the cellular membrane and eNOS instead of the Ang II-NADPH oxidase pathway.

## 1. Introduction

Asymmetric (N^G^, N^G^) dimethylarginine (ADMA), a naturally occurring L-Arginine (ARG) analog, is a methylated amino acid derived from the proteolysis of proteins [1–3]. ADMA has been associated with impaired endothelial function in humans [4], and clinical evidence suggests that serum ADMA may be a novel cardiovascular risk factor [5,6]. Elevated ADMA plasma concentrations have been demonstrated in patients with a diverse array of diseases, including hypertension [7], hyperlipidemia [8], hyperhomocyst(e)inemia [9], coronary artery disease [10], peripheral arterial occlusive disease [11], congestive heart failure [12], stroke [13], pulmonary hypertension [14], and end-stage renal disease [15].

Several studies have demonstrated that ADMA induces oxidative stress in vascular tissues. Veresh *et al.* [16] showed that 1 mM ADMA increased dihydroethidium (DHE) fluorescence in isolated rat femoral artery. Superoxide (O_2_^•−^) dismutase reversed the deleterious vascular effects of ADMA and ethidium bromide fluorescence [17]. Serum ADMA was correlated, in multiple linear regression, with vascular O_2_^•−^ levels in the saphenous veins and internal mammary arteries taken from 201 patients undergoing coronary bypass surgery [18].

The mechanisms by which ADMA induces vascular oxidative stress have not been completely defined. Results from chronic administration of ADMA in mice appeared to indicate that renin-angiotensin system (RAS) may be involved [19–22]. Recently, Veresh *et al.* [16] showed that in isolated rat arterioles, ADMA activates the local RAS, releasing angiotensin II (Ang II), which in turn activates NADPH oxidase, leading to O_2_^•−^ accumulation. However, Antoniades *et al.* [23] found no correlation between elevated serum ADMA and NADPH-stimulated vascular O_2_^•−^. Thus, the exact role of NADPH oxidase in mediating ADMA-induced vascular O_2_^•−^ accumulation is still unclear.

Examination of the mechanisms of ADMA-induced oxidative stress in cell culture systems, particularly in human vascular endothelial cells, has been quite limited. In preliminary studies (*n* = 3), Antoniades *et al.* [23] showed that incubation of human umbilical vein endothelial cells (HUVEC) with 1 mM for 48 h induced a 2-fold increase in O_2_^•−^ accumulation. However, serum ADMA concentrations are typically below 1 μM, and the relationship between O_2_^•−^ induction and ADMA concentration was not determined.

Here, we examined the oxidative stress effects of ADMA using HUVEC. We show that the behavior of ADMA-induced DHE fluorescence is significantly different to that of Ang II, and that ADMA-induced oxidative stress requires the participation of both the cationic transport system in the cellular membrane, and endothelial nitric oxide synthase (eNOS). Evidence for ADMA-induced eNOS uncoupling and involvement of tetrahydrobiopterin (BH_4_) is presented.

## 2. Results and Discussion

### 2.1. ADMA Induces Enchanced DHE Fluorescence in HUVEC Cells and Cell Membranes

**Figure 1:**
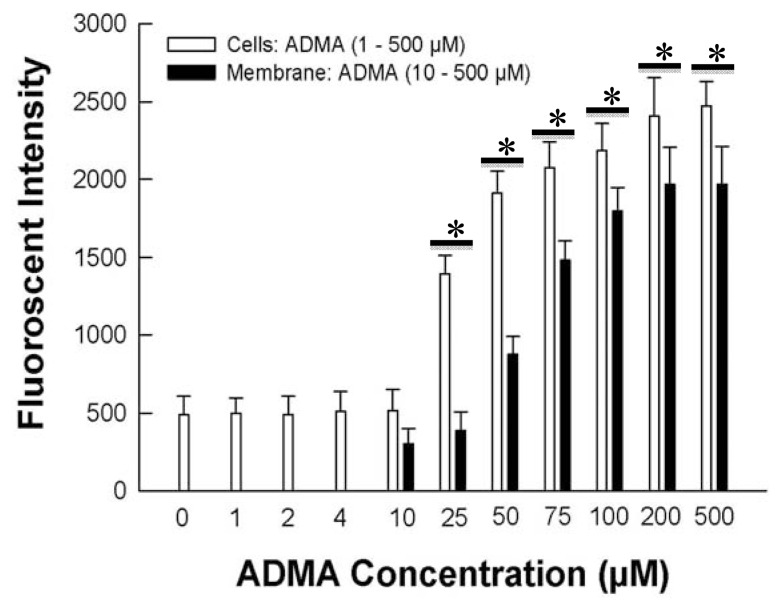
Dihydroethidium (DHE) fluorescence in human umbilical vein endothelial cells (HUVEC) whole cells incubated chronically for 7 days with 0 to 500 μM asymmetric dimethylarginine (ADMA), or HUVEC membranes incubated for 30 min with 10 to 500 μM ADMA. * *p* < 0.05 versus 10 μM ADMA whole cell or cell membrane treatment. *n* = 6.

Upon exposure to ADMA at various concentrations above 10 μM for 7 days, HUVEC displayed a concentration-dependent increase in DHE fluorescence intensity (Figure 1), which was attained near plateau values over 100 μM ADMA. No increase in DHE fluorescence was observed below 10 μM ADMA. This concentration-dependency was reproduced by incubating the HUVEC cell membranes for 30 min, indicating that the cell membrane was the principal cellular sites for ADMA to produce oxidative stress, and that intracellular proteins are not critical for this effect (Figure 1).

Although we have shown 10 μM ADMA to be the threshold for measuring sufficient DHE fluorescence, our results do not necessarily mean that ADMA would not produce effects of oxidative stress in cells below this concentration, because we measured DHE fluorescence only at one time point, and that certain cellular proteins could have a higher sensitivity toward smaller changes in cellular oxidative stress which our chemical assay system could not detect. Zhao *et al.* [24] showed that DHE fluorescence cannot be equated quantitatively to O_2_^•−^ production. Thus, the enhanced DHE fluorescence that we observed may include other reactive oxygen species besides O_2_^•−^. However, using the same methods and conditions, we showed in a parallel study [25] that L-arginine induced DHE fluorescence was completely inhibited by PEG-superoxide dismutase, indicating that this oxidative stress most likely involved the production of O_2_^•−^. Our current results also showed that, when intact HUVEC were incubated with 100 μM ADMA for 7 days, the enhancement in DHE fluorescence was similar in magnitude to that observed for L-arginine under identical conditions [25].

### 2.2. Involvement of Membrane ADMA Transport and eNOS in Oxidative Stress

**Figure 2:**
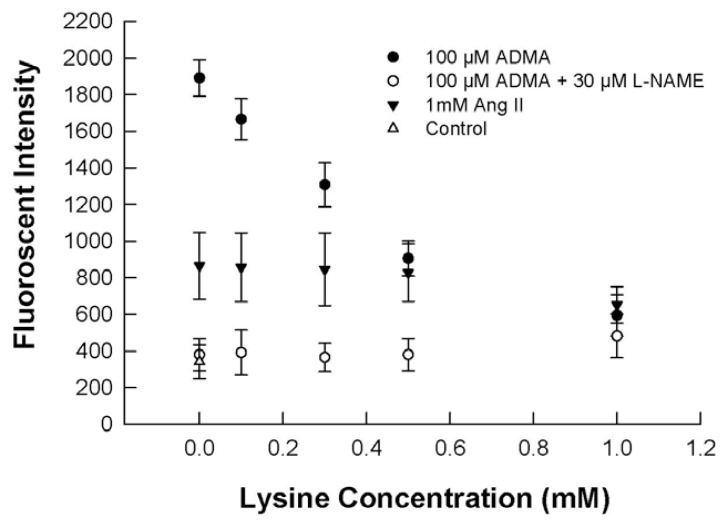
Effects of L-lysine and L-nitroarginine methyl ester (L-NAME) on DHE fluorescence production from HUVEC cell membranes after exposure to 100 μM ADMA or 1 mM Ang II for 30 minutes. *n* = 6.

Co-incubation of 100 μM ADMA with L-lysine (0.1 to 1 mM) reduced DHE fluorescence from isolated HUVEC cell membranes in a concentration-dependent manner, and the increase in DHE fluorescence was completely abolished at 1 mM L-lysine, which utilizes the cellular transport system for basic amino acids including ADMA. In contrast, L-lysine had no effect on the extent of oxidative stress induced by Ang II (Figure 2). An inhibitor of eNOS, L-nitroarginine methyl ester (L-NAME), at 30 μM, completely inhibited the DHE fluorescence of ADMA on HUVEC cell membranes, at all L-lysine concentrations (Figure 2), and the fluorescence observed was not different from that of control (*i.e.*, no ADMA).

These results indicate that the oxidative effects of ADMA requires cellular uptake, probably via its principal transport protein, Cationic Amino Acid Transporter 1 (CAT-1), which ADMA shares with ARG and L-lysine [26]. Since L-lysine, up to 1 mM, did not affect DHE fluorescence induced by Ang II, it indicates that this amino acid did not exert a general anti-oxidant effect over the concentration range studied, and that ADMA and Ang II may induce oxidative stress via different mechanisms. Figure 2 also shows that 30 μM L-NAME completely inhibited DHE fluorescence by ADMA, whether or not L-lysine was present. This result indicates that the oxidative mechanism of ADMA may involve the participation of eNOS and its possible uncoupling to produce O_2_^•−^ [18].

### 2.3. Effect of BH_4_ on ADMA-Induced Oxidative Stress

**Figure 3:**
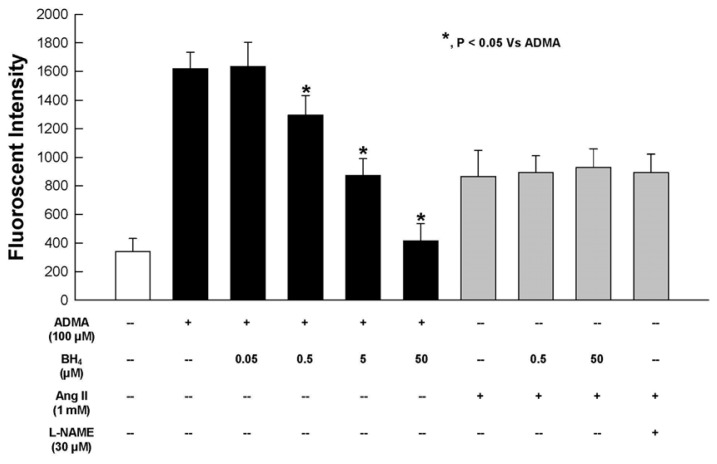
Effect of BH_4_ on ADMA or Ang II-induced DHE fluorescence from isolated HUVEC membranes after incubation for 30 minutes. *n* = 6.

Addition of BH_4_, a critical cofactor for eNOS activity, significantly reduced the DHE fluorescence intensity induced by 100 μM ADMA on HUVEC cell membranes, and at 50 μM BH_4_, the oxidative effects of ADMA was completely abrogated. In contrast, BH_4_, up to 50 μM, had no effect on Ang II-induced DHE fluorescence. These results indicate the protective effects of BH_4_ against oxidative stress (Figure 3) during ADMA supplementation. Again, in contrast, BH_4_ did not act as a general antioxidant because it did not attenuate the DHE fluorescence from Ang II, even at a high concentration of 50 μM. Electromagnetic resonance studies showed that in the absence of BH_4_, O_2_^•−^ is the sole *in vitro* product of recombinant eNOS [27]. In the absence of BH_4_, electron transfer within eNOS becomes uncoupled and ferrous dioxygen releases O_2_^•−^ with a finite probability [27].

### 2.4. Nitric Oxide (NO) Bioavailability

**Figure 4:**
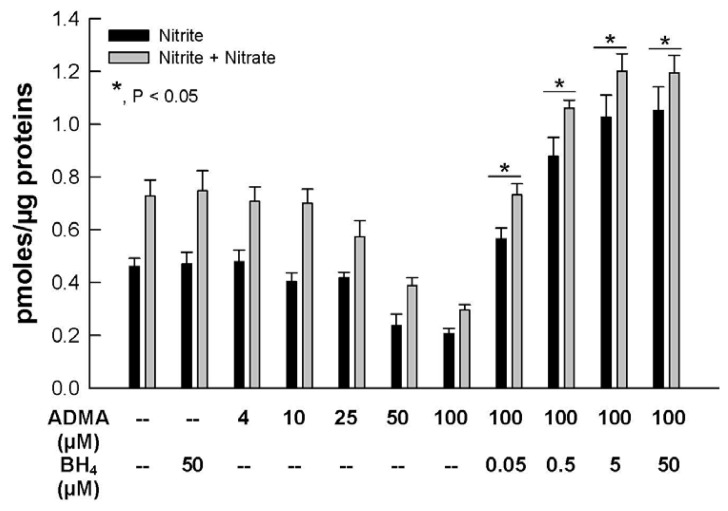
Effects of BH_4_ on NO_2_^−^ or nitrite/nitrate (NO*_x_*) production in the presence of ADMA. HUVEC NO were exposed for 2 hrs in 0–100 μM ADMA alone or 100 μM ADMA plus 0.05 to 50 μM BH_4_. *, *p* < 0.05 versus 100 μM ADMA treatment. *n* = 6.

NO bioavailability was assessed by determining the accumulation of inorganic nitrite and total nitrite/nitrate ions (Figure 4). Similar to the concentration-dependent effect of ADMA on DHE fluorescence (Figure 1), nitrite and nitrite/nitrate production were not increased by ADMA concentrations up to 10 μM. However, above this concentration, nitrite and nitrite/nitrate accumulation was significantly decreased by ADMA. However, adding BH_4_ to the cells not only reversed the diminished production of NO from ADMA, but enhanced NO availability as BH_4_ concentration increased. An earlier report by Vasquez-Vivar and colleagues [28] provided evidence that increase in oxidation of BH_4_ produced BH_2_ induces eNOS uncoupling and decreased NO production. These results indicate that ADMA decreased NO formation most likely through increased oxidative stress, which in turn brought about eNOS uncoupling. The beneficial effect of BH_4_ on cellular NO availability in the presence of ADMA is highly consistent with this interpretation.

Closs *et al.* [29] demonstrated that ADMA is a good substrate for human CAT. The capacity and activity of CATs determines the partition of cationic amino acids across cells and thereby the ratio of ARG to ADMA in competition for the eNOS binding site. Here, we showed in Figure 4 that in the absence of extracellular ARG, ADMA concentration-dependently inhibited cellular nitrite and nitrite/nitrate production. This result could be attributed, at least in part, by the displacement of endogenous ARG from eNOS in the presence of increasing ADMA concentrations. However, the strong reversing effect of BH_4_ (Figure 4) argued more strongly on behalf of a mechanism of oxidative stress, rather than mere displacement of ARG from eNOS binding, for the reduced NO availability induced by ADMA.

Our results indicate that ADMA behaved differently to Ang II in generating cellular NO. As expected, oxidative stress brought about by Ang II was unaffected by L-NAME, L-lysine and BH_4_ (Figures 2,3), while these substances significantly affect ADMA-induced oxidative stress.

### 2.5. Effects of Apocynin or Losartan on ADMA-Induced O_2_^•−^ Production

A role for NADPH oxidase for mediating ADMA O_2_^•−^ formation was implicated recently [16] by the observation that the NADPH oxidase inhibitor (apocynin) or the Ang II receptor inhibitor (losartan) reduced O_2_^•−^ production from 100 μM ADMA. However, in this study, effects from these agents by themselves, *i.e.*, 300 μM apocynin or 20 μM losartan alone without ADMA, were not investigated. HUVEC when exposed to 100 μM ADMA exhibited increased DHE fluorescence signals (1989 ± 134 arb units) *vs.* control cells (599 ± 114 arb units, *p* < 0.05). These increases were abrogated in the presence of 300 μM apocynin (660 ± 141 arb units) or 20 μM lorsatan (683 ± 113 arb units). However, 300 μM apocynin alone, or 20 μM lorsatan alone, significantly reduced DHE fluorescence signals to about half of the control value (*i.e.*, to 338 ± 62 and 337 ± 71 arb units, respectively). The effect of apocynin observed here is consistent with its general antioxidant properties [30]. While these NADPH oxidase-related agents did reduce the production of DHE fluorescence from ADMA, the effect is contributed by a reduction in baseline production or a general antioxidant effect. Thus, a conclusion of NADPH oxidase involvement in ADMA-induced oxidative stress cannot be conclusively proven by the use of these inhibitors alone.

## 3. Experimental Section

### 3.1. Supplies and Reagents

Endothelial cell culture was purchased from American Type Culture Collection (Manassas, VA) and culture reagents were from Invitrogen (Carlsbad, CA). All culture supplies and chemicals were from Laboratory Product Sales (Rochester, NY) and Sigma-Aldrich (St. Louis, MO) respectively. The nitrite-nitrate fluorometric assay kit was purchased from Cayman Chemical Company (Ann Arbor, MI). Deionized water (18 MΩ) was used in all experiments.

### 3.2. Cell Studies

HUVEC were cultured in physiological F-12K medium containing 100 μM ARG and 90 mg/dL glucose, supplemented with 20% horse serum, 100 U/mL penicillin and 100 μg/mL streptomycin. Cells were maintained in a humidified chamber at 37 °C with 5% CO_2_, and passages between 6 and 16 (mean passage number = 9 ± 3) were used in all the experiments. For acute studies, cell culture in 6-well plates were incubated in Locke’s buffer containing either 12.5 to 500 μM ADMA, or combinations of 100 μM ADMA with or without 300 μM apocynin, 10 μM losartan, or 0.05 to 50 μM BH_4_ for 2 h. Chronic ADMA effect was assessed by incubating cultured cells in F-12K medium containing 1 to 500 μM ADMA for 7 days.

### 3.3. Membrane Studies

HUVECs were washed twice with 2 mL of phosphate buffered saline (PBS) and incubated with trypsin EDTA (0.5 mL) for less than 3 min before adding equal amount of F-12K medium (0.5 mL). The cells were centrifuged at 300 *g* for 5 min, washed twice with 1 mL of PBS, resuspended in 1 mL Locke’s buffer, sonicated for 2 min, and centrifuged at 300 *g* for 5 min to separate the membrane fractions as pellets. The membrane fractions were reconstituted in 1 mL Locke’s buffer containing either 12.5 to 500 μM ADMA, or combinations of 100 μM ADMA with or without L-lysine (0.1–1 mM), 30 μM L-NAME, 1 mM angiotensin II (Ang II), or BH_4_ (0.05–50 μM), and incubated for 30 min for subsequent determination of DHE fluorescence or nitrite/nitrate.

### 3.4. DHE Fluorescence Measurement Using a Micro-Plate Reader

Oxidative stress in HUVEC and its membrane fractions was assessed by DHE fluorescence [24]. At the end of the incubations, cells or membrane fractions were washed and incubated in Locke’s buffer at a final DHE concentration of 10 nM for 20 min. The resulting mixtures were harvested in acetonitrile (0.2 mL/well), sonicated (10 s), and centrifuged (13,000 *g* for 5 min at 4 °C). The supernatant fraction was air-dried, reconstituted in PBS and fluorescence was determined, in duplicate, using a micro-plate reader at excitation and emission wavelengths of 490 and 570 nm, respectively.

### 3.5. Inorganic Nitrite and Total Nitrite/Nitrate Determination

Cell lysate samples or freshly prepared nitrite standard were first brought to volume of 100 μL with double-deionized water. Samples were protected from light, and 10 μL of freshly prepared diaminonaphalene (DAN, 0.05 mg/mL in 1 M HCl) was added and mixed immediately. After 10 min incubation at room temperature, the reaction was terminated with 5 μL of 2.8 M NaOH. The intensity of the fluorescent signal produced was measured using a plate reader with excitation at 360 nm and emission read at 420 nm, with a gain setting at 100%.

In order to measure total nitrite/nitrate, nitrate was converted to nitrite by the action of nitrate reductase from *Aspergillus niger*. Briefly, the samples were incubated with 40 μM NADH and 14 mU of enzyme in a final volume of 50 μL of 20 mM Tris, pH 7.6, followed by 30 min incubation with 10 μL of DAN at room temperature. The reaction was terminated after 30 min with 20 μL of NaOH. NO*_x_* in the samples were then calculated by first subtracting the value of the enzyme blank containing NADH. The values were further normalized using total protein concentration, which were measured according to the Lowry method [31] using bovine serum albumin as standard.

### 3.6. Statistical Analysis

Data are presented as mean ± standard deviation (*n* = 4 replicates) unless otherwise stated. Statistical comparisons among groups were performed using one-way analysis of variance (ANOVA), followed by Fisher’s and Tukey’s post-hoc test procedure (version 15.x; Minitab). Statistical significance was concluded when *p* < 0.05.

## 4. Conclusions

Our studies utilized HUVEC to explore the mechanisms by which ADMA generates oxidative stress. In whole cells and isolated cell membranes, ADMA increased cellular DHE fluorescence at concentrations above 10 μM, indicating that intracellular proteins are not critical for this action. ADMA-induced DHE fluorescence was inhibited by L-lysine and L-NAME, suggesting that cellular uptake and interaction with eNOS were necessary, consistent with the mechanisms of oxidative stress induced by ARG that we have shown recently [25]. Addition of BH_4_ abrogated ADMA-induced cellular oxidative stress and reduced NO availability, indicating that the oxidative effects of ADMA may be mediated via eNOS uncoupling. In contrast, Ang II-induced DHE fluorescence in HUVEC was not affected by L-lysine, L-NAME and BH_4_. These results suggest that ADMA most likely induced oxidative stress through the CAT-1/eNOS complex, instead of the RAS, as has been previously suggested.
